# Expression of CSTF2 in oral squamous cell carcinoma and its relationship with immune infiltration and poor prognosis

**DOI:** 10.3389/froh.2025.1548829

**Published:** 2025-02-07

**Authors:** Zumulaiti Aierken, Muertiza Muhetaer, Zhang Lei, Ainiwaerjiang Abudourousuli

**Affiliations:** ^1^ Department of Stomatology, The First People’s Hospital of Kashi Prefecture, Kashi, Xinjiang, China; ^2^ Department of Pathology, The First People’s Hospital of Kashi Prefecture, Kashi, Xinjiang, China; ^3^ Kashi Prefecture Cancer Research Institute, The First People’s Hospital of Kashi Prefecture, Kashi, Xinjiang, China

**Keywords:** oral squamous cell carcinoma, CSTF2, prognosis, immune infiltration, differential expression, biomarker

## Abstract

**Background:**

Oral squamous cell carcinoma (OSCC) is a prevalent and devastating malignancy of the oral cavity that profoundly affects patient survival and quality of life (QOL). Cleavage Stimulation Factor Subunit 2 (CSTF2) is known to influence tumor development across multiple cancer types. However, its specific association with patient prognosis and immune cell infiltration in OSCC remains insufficiently understood.

**Methods:**

To assess the expression levels and prognostic implications of CSTF2 in OSCC, comprehensive data were acquired from The Cancer Genome Atlas (TCGA) and subsequently normalized. Immunohistochemical staining of tissue microarrays was performed to analyze CSTF2 expression in the OSCC samples. Differences in CSTF2 expression between OSCC and adjacent non-cancerous samples were evaluated using the Wilcoxon rank-sum test. Functional enrichment analyses have been performed to identify biological pathways and functions associated with CSTF2. The relationship between the infiltration of various immune cells and CSTF2 expression levels was assessed using single-sample gene set enrichment analysis (ssGSEA). Ultimately, the prognostic significance of CSTF2 was evaluated through Kaplan–Meier survival analysis, in conjunction with univariate and multivariate Cox regression analyses, as well as receiver operating characteristic (ROC) curves.

**Results:**

High CSTF2 expression was observed in OSCC and associated with unfavorable clinicopathological variables, including histological grade and lymphnode neck dissection. Functional enrichment analysis indicated that CSTF2 plays a role in epidermal development and differentiation, immunoglobulin complexes, peptidases and endopeptidase inhibitor activity, and cytochrome P450 metabolic processes. Additionally, the overexpression of CSTF2 exhibited a negative correlation with the infiltration of immature dendritic cells (iDCs), cytotoxic cells, and plasmacytoid dendritic cells (pDCs). Notably, elevated CSTF2 expression is significantly associated with reduced patient outcomes.

**Conclusion:**

Elevated CSTF2 expression in OSCC is associated with poor prognostic outcomes, highlighting its capacity to function as an innovative prognostic biomarker and a target for therapeutic interventions.

## 1 Introduction

Oral squamous cell carcinoma (OSCC) is a common and devastating malignancy of the oral cavity that severely affects both the quality of life and survival rates of those diagnosed (1). It accounts for a significant proportion of head and neck cancers and is characterized by an aggressive clinical behavior and a propensity for local invasion and regional metastasis (2). Chronic exposure to various risk factors, such as cigarette smoking, alcohol, betel quid (BQ), and human papillomavirus (HPV), may result in the emergence of oral potentially malignant disorders (OPMDs), which are oral mucosal lesions associated with a heightened risk of progressing to OSCC (3–5). Despite improvements in conventional treatment strategies, such as surgery, chemotherapy and radiotherapy, the overall survival rate of individuals diagnosed with OSCC remains approximately 40% (6). Early detection and accurate prognostic assessment are crucial for improving patient outcomes (7). However, current diagnostic approaches, which primarily rely on clinical examination and histopathological evaluation, often fail to identify the disease in its early stages (8). Current research has identified several biomarkers associated with OSCC; however their clinical utility often varies because of the heterogeneity of the disease (9). Furthermore, studies have indicated that other biological markers, such as CD44, may serve as indicators of malignant transformation in oral epithelial dysplasia and provide prognostic information regarding tumor behavior (10). However, the specificity and sensitivity of these markers in predicting clinical outcomes in patients with OSCC remain unclear. Consequently, there is a pressing need for novel research to uncover the molecular mechanisms underlying OSCC, and to identify reliable prognostic biomarkers that could aid clinical decision-making and enhance patient prognostication.

CSTF2, which is located on chromosome 19, is crucial for pre-mRNA processing and polyadenylation (11). As a component of the cleavage and polyadenylation machinery, CSTF2 influences gene expression by regulating mRNA transcript stability and maturation of mRNA transcripts (12). Dysregulation of CSTF2 has been implicated in multiple cancers, including hepatocellular carcinoma (HCC), lung cancer (LUAD), and its expression levels are associated with tumor advancement and unfavorable survival rates (13). Elevated CSTF2 expression levels may contribute to increased cell proliferation and survival, further complicating cancer outcomes (14). However, the clinical significance of CSTF2 and its correlations with immune cell infiltration in OSCC remains unclear.

In the present study, the expression levels and prognostic relevance of CSTF2 in OSCC were elucidated via bioinformatics and tissues microarray techniques. Our results indicated that CSTF2 is substantially overexpressed in OSCC and correlated with negative clinicopathological characteristics, modified immune infiltration and unfavorable prognostic outcomes. In summary, CSTF2 may serve as a promising biomarker for both diagnostic and theraputic strategies for OSCC.

## 2 Methods

### 2.1 Data acquisition

Clinical and transcriptomic data from a total of 362 human oral epithelial tissue samples, consisting of 330 OSCC samples and 32 non-tumor tissues, were sourced from TCGA. The Fragments Per Kilobase of transcript per Million mapped reads (FPKM) format data were transformed into transcripts per million (TPM) for the analytical procedures.

### 2.2 Tissue microarray

The tissue microarray utilized in this study was provided by Zhongke Guanghua (Xi'an) Intelligent Biological Co., Ltd. (catalog number: HN0580c01), ethics approved by same communicated with number: Csyayj2024081. The array included 39 cancer tissues and 19 adjacent non-cancerous tissues(2 tissues of buccal mucosa, 14 tissues of tongue and 3 tissues of glossooharyngeal mucosa), enabling a comprehensive assessment of CSTF2 expression across differing tumour environments.

### 2.3 Immunohistochemistry

Tumor and adjacent non-cancerous tissues were preserved using 10% formalin, subsequently paraffin embedded, then sectioned into 4–6 *μ*m slices. Following deparaffinization, rehydration, and microwave antigen retrieval, the slides were incubated with a 1:200 dilution of anti-CSTF2 (Bioss, #A8116) at 4°C. After 12 h, the slides were subjected to a 30 min exposure to a secondary antibody at room temperature, followed by 3,3′-Diaminobenzidine (DAB) substrate staining and hematoxylin counterstaining.

### 2.4 Assessment of immunohistochemical staining

Two pathologists, who were not associated with this project, assessed the pathological slides according to the latest literature (15, 16). The diagnosis were based on the criteria defined in the 2022 WHO classification of head and neck tumors (17). The tissues were scored on a scale of 0, 1, 2, and 3, where 0 represented no expression, 1 indicated low expression, 2 represented moderate expression, and 3 indicated high expression. Cohen` kappa test was performed to confirm the results.

### 2.5 Expressed Gene Analysis

Using the median expression levels of CSTF2, patients with OSCC (*n* = 330) were categorized into two distinct groups to ensure a clear differentiation in biological response. The R package “Limma” was employed to discern differentially expressed genes (DEGs) between cancer and non-cancerous samples, applying thresholds of an adjusted *p*-value < 0.05 and |log2-fold-change (FC)| >1.5 for significance. The association between CSTF2 and the top ten DEGs was analyzed using Spearman's correlation method. R package “GOplot” (v 1.0.2) was used to applied Gene Ontology (GO) to classify DEGs into various categories. Furthermore, GSEA analysis was executed via the “ClusterProfiler” R package for identifying enriched biological pathways associated with CSTF2.

### 2.6 Immune infiltration analysis

The levels of immune infiltration for 24 distinct immune cell types were computed, and their relative enrichment scores in OSCC were assessed using single-sample gene set enrichemnt analysis (ssGSEA). The association between CSTF2 expression and the infiltration of various immune cells in OSCC were evaluated via the Spearman's correlation test. Differences in immune infiltration levels between high and low CSTF2 expression groups were evaluated using the Wilcoxon rank-sum test, providing insights into the immunological landscape associated with CSTF2 expression in OSCC.

### 2.7 Prognostic assessment of CSTF2 in OSCC

The influence of CSTF2 expression in OSCC patients was evaluated using the Kaplan–Meier method. The potential independent prognostic value of CSTF2 in OSCC were assessed via univariate and multivariate Cox regression analyses. The diagnostic and prognostic efficacy CSTF2 in OSCC and various cancer types were assessed via receiver operating characteristic (ROC) curves. Furthermore, the univariate Cox regression analysis has been used to explore the interrelations among the variables in the prognostic model. Regplot, survival, and RMS packages were used to analyze clinicopathological features and risk scores.

### 2.8 Statistical analysis

The Wilcoxon rank-sum test was employed to facilitate comparisons between two distinct groups, whereas the Spearman correlation test was used to investigate the relationships among various variables. Furthermore, both univariate and multivariate Cox proportional hazard regression analyses were performed to determine the prognostic factors. All statistical analyses were bilateral, with *p*-values < 0.05 considered statistically significant, thereby guaranteeing the integrity of the results.

## 3 Results

### 3.1 CSTF2 is significantly overexpressed in OSCC

**Figure 1:**
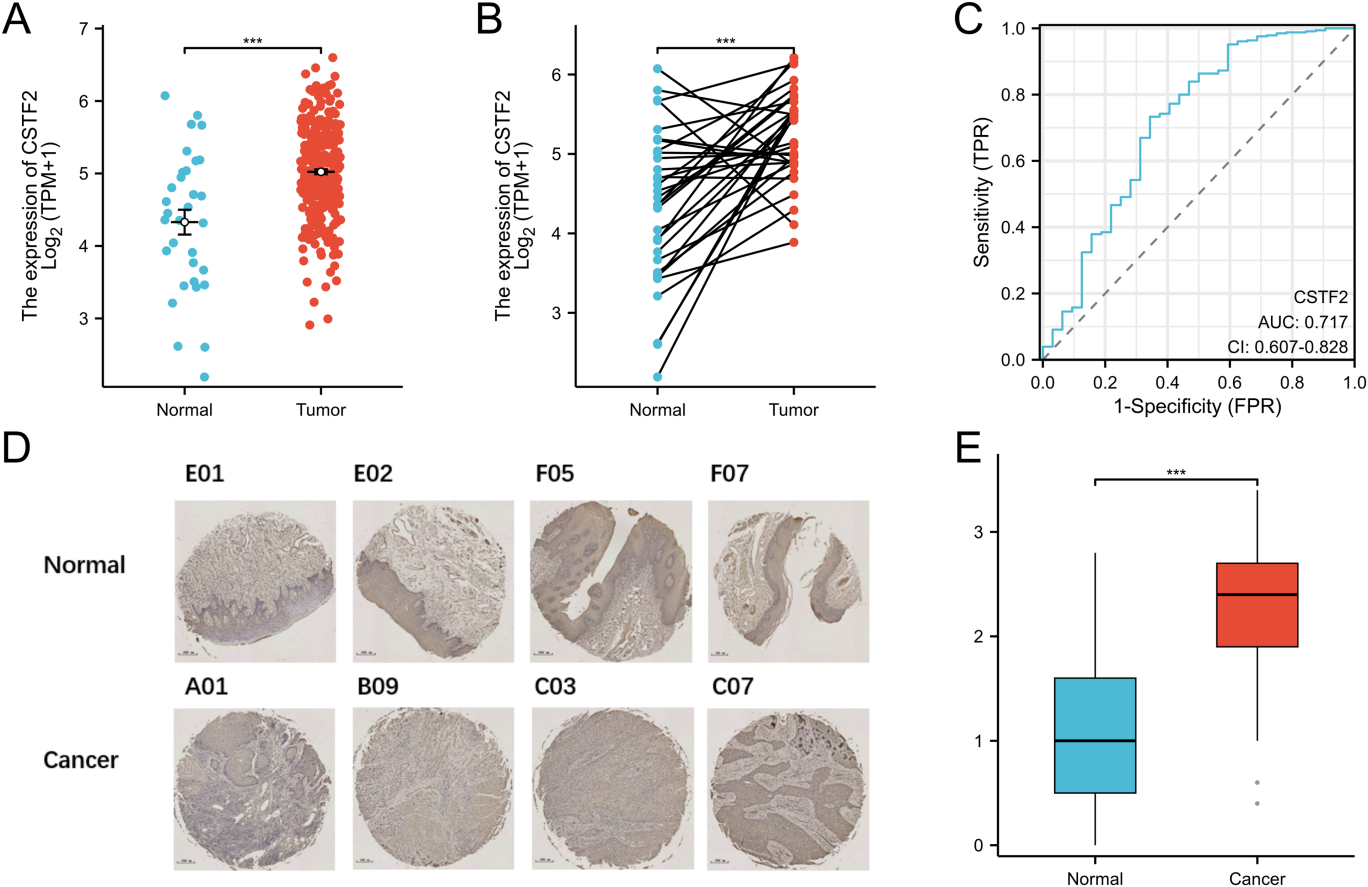
CSTF2 is significantly overexpressed in OSCC. **(A)** CSTF2 expression in TCGA-OSCC. **(B)** CSTF2 expression in paired OSCC samples. **(C)** ROC curve of CSTF2 in OSCC. **(D)** Immunohistochemical staining of CSTF2 in OSCC tissues chip. **(E)** Quantification of CSTF2 immunohistochemical staining.

We evaluated the expression levels of the CSTF2 in OSCC tissues relative to those in adjacent non-cancerous tissues. Our Statistical analysis showed high CSTF2 expression in OSCC samples compared to that in the adjacent non-cancerous tissues (Figure 1A). This differential expression was confirmed in 32 paired OSCC samples (Figure 1B). An ROC curve was generated to assess CSTF2's diagnostic potential, resulting in an AUC of 0.717, indicating significant accuracy in distinguishing OSCC from adjacent tissues (Figure 1C). In addition, tissue microarray analysis coupled with immunohistochemistry was performed to validate of CSTF2 expression. Immunohistochemistry results revealed that CSTF2 was predominantly localized in the cell cytoplasm, with partial expression in the nucleus and no expression in the cell membrane. Our analyses further confirmed that the expression levels of CSTF2 were notably elevated in OSCC tissues compared to the adjacent non-cancerous tissues (Figure 1D, Supplementary Figure S1). Quantitative analysis revealed significantly increased staining intensity in OSCC cells, with Cohen's kappa index score of 0.794 (Figure 1E). The correlations between CSTF2 expression and the clinical features of OSCC patients are analyzed (Tables 1a, 1b). Statistical analyses showed that variables, such as age, sex and histological grade were correlated with CSTF2 expression. Logistic regression further identified significant correlations between CSTF2 expression and age and gender (Table 2). Together, these findings suggest that CSTF2 is significantly overexpressed in OSCC and linked to various clinicopathological factors.

**Table 1a:** The epidemiological features of OSCC patients.

Characteristics	CSTF2-Low	CSTF2-High	*P*
*N*	165	165	
Age, *n* (%)			0.024^a^*
<= 60	68 (20.7%)	88 (26.7%)	
>60	97 (29.5%)	76 (23.1%)	
Gender, *n* (%)			0.004^a^*
Male	102 (30.9%)	126 (38.2%)	
Female	63 (19.1%)	39 (11.8%)	
Anatomic neoplasm subdivision, *n* (%)			0.858^b^
Alveolar Ridge	9 (2.7%)	9 (2.7%)	
Base of tongue & Oral Tongue	72 (21.8%)	78 (23.6%)	
Buccal Mucosa	11 (3.3%)	11 (3.3%)	
Floor of mouth	35 (10.6%)	26 (7.9%)	
Hard Palate	4 (1.2%)	3 (0.9%)	
Oral Cavity	34 (10.3%)	38 (11.5%)	
Alcohol history, *n* (%)			0.360^a^
No	57 (17.7%)	48 (14.9%)	
Yes	106 (32.9%)	111 (34.5%)	
Smoker, *n* (%)			0.803^a^
No	43 (13.3%)	45 (13.9%)	
Yes	119 (36.7%)	117 (36.1%)	

^a^ Represents Chisq test.

^b^ Represents Yates' correction test.

* Statistically significant difference (*p* < 0.05).

**Table 1b:** The clinicopathological features of OSCC patients.

Characteristics	CSTF2-Low	CSTF2-High	*P*
*n*	165	165	
Pathologic stage, *n* (%)			0.374^a^
Stage I	12 (4%)	5 (1.7%)	
Stage II	26 (8.7%)	28 (9.4%)	
Stage III	30 (10%)	31 (10.4%)	
Stage IV	81 (27.1%)	86 (28.8%)	
Pathologic T stage, *n* (%)			0.384^a^
T1	18 (5.9%)	11 (3.6%)	
T2	45 (14.8%)	55 (18%)	
T3	35 (11.5%)	31 (10.2%)	
T4	53 (17.4%)	57 (18.7%)	
Pathologic N stage, *n* (%)			0.400^b^
N0	63 (22.8%)	55 (19.9%)	
N1	24 (8.7%)	26 (9.4%)	
N2	50 (18.1%)	56 (20.3%)	
N3	0 (0%)	2 (0.7%)	
Histologic grade, *n* (%)			0.010^b^*
G1	36 (11.2%)	16 (5%)	
G2	100 (31.1%)	101 (31.4%)	
G3	28 (8.7%)	39 (12.1%)	
G4	0 (0%)	2 (0.6%)	
Lymphovascular invasion, *n* (%)			0.741^a^
No	83 (34.6%)	82 (34.2%)	
Yes	36 (15%)	39 (16.2%)	
Lymphnode neck dissection, *n* (%)			0.630^a^
No	21 (6.4%)	24 (7.3%)	
Yes	143 (43.6%)	140 (42.7%)	
Radiation therapy, *n* (%)			0.142^a^
No	54 (18.3%)	62 (21%)	
Yes	99 (33.6%)	80 (27.1%)	

^a^ Represents Chisq test.

^b^ Represents Yates' correction test.

* Statistically significant difference (*p* < 0.05).

**Table 2:** The examination of CSTF2 via logistic regression analysis in OSCC.

Characteristics	Total (*N*)	OR (95% CI)	*P* value
Age (>60 vs. <=60)	329	0.605 (0.391–0.937)	**0** **.** **024** *
Gender (Male vs. Female)	330	1.995 (1.238–3.215)	**0**.005*
Pathologic stage (Stage III & Stage IV vs. Stage I & Stage II)	299	1.214 (0.712–2.070)	0.477
Pathologic T stage (T3 & T4 vs. T1&T2)	305	0.955 (0.606–1.504)	0.841
Pathologic N stage (N2 & N3 vs. N0 & N1)	276	1.246 (0.768–2.022)	0.374
Histologic grade (G3 & G4 vs. G1 & G2)	322	1.702 (0.992–2.922)	0.054
Alcohol history (Yes vs. No)	322	1.244 (0.779–1.984)	0.361
Smoker (Yes vs. No)	324	0.939 (0.576–1.533)	0.803
Lymphovascular invasion (Yes vs. No)	240	1.097 (0.635–1.893)	0.741
Lymphnode neck dissection (Yes vs. No)	328	0.857 (0.456–1.609)	0.630
Radiation therapy (Yes vs. No)	295	0.704 (0.440–1.125)	0.142

* Statistically significant difference (*p* < 0.005).

### 3.2 Functional enrichment analyses of CSTF2-related DEGs in OSCC

**Figure 2:**
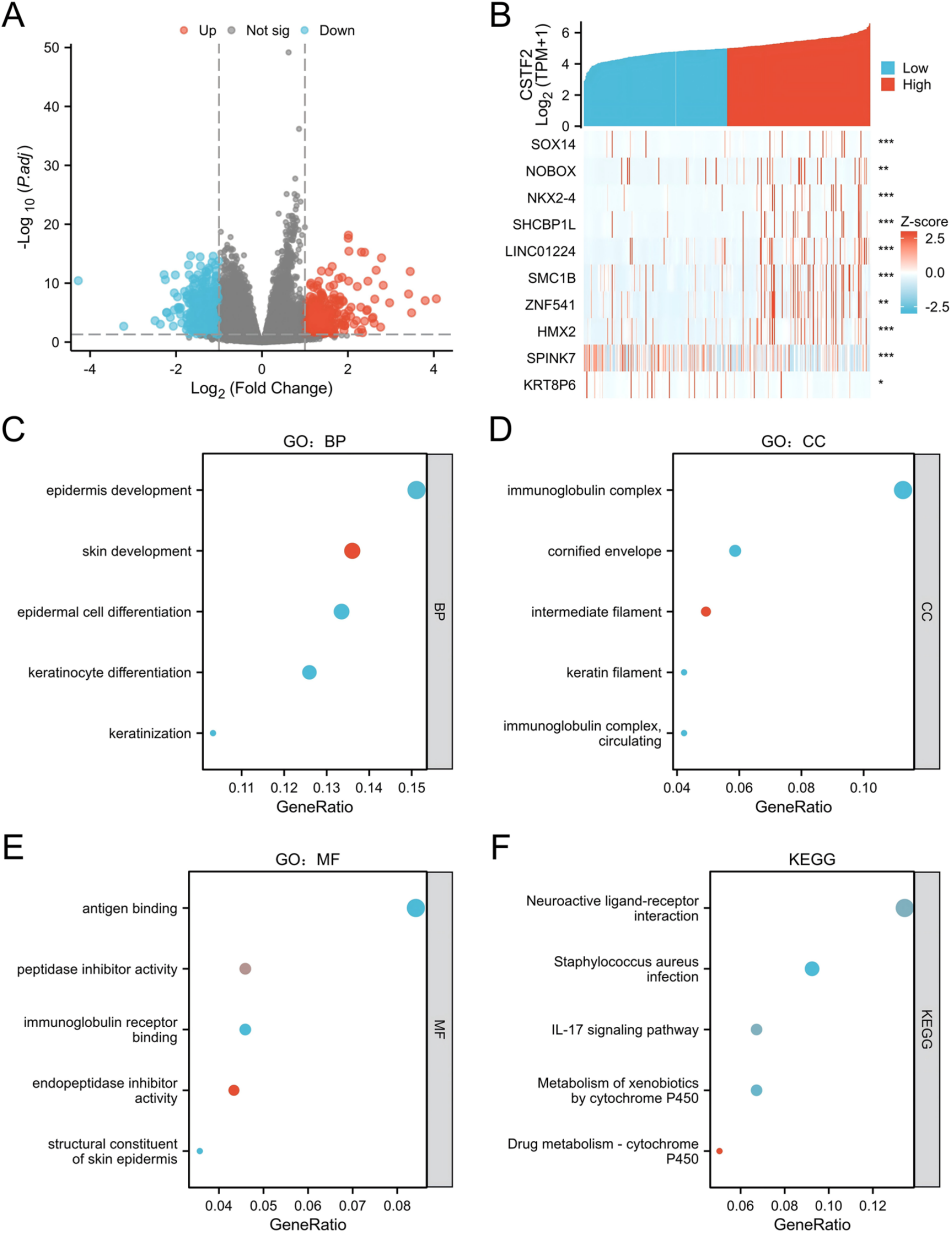
Examination of CSTF2-related DEGs and their functional enrichment in OSCC. **(A)** Volcano plot illustrating DEGs, with significantly downregulated (blue) and upregulated (red) genes. **(B)** Heatmap showing the correlation between CSTF2 expression and the top ten DEGs. **(C)** GO enrichement analysis for biological process (GO-BP) of DEGs. **(D)** GO enrichement analysis for cellular component (GO-CC) of DEGs. **(E)** GO enrichement analysis for molecular functions (GO-MF) of DEGs. **(F)** KEGG analysis of DEGs.

CSTF2 related differentially expressed genes (DEGs) were examined to further understand the biological significance of CSTF2 in OSCC. A cohort of 330 OSCC patients was stratified into CSTF2-high and CSTF2-low expression groups using the average CSTF2 expression level as a criterion. Comparative analysis identified 895 DEGs, with 496 downregulated genes and 399 upregulated genes, exhibiting an absolute log-fold change exceeding 1.5 and applying thresholds of an adjusted *p*-value < 0.05. A volcano plot shows the DEGs (Figure 2A). Figure 2B depicts the association between CSTF2 and the top ten DEGs, including SOX14, NOBOX, NKX2–4, SHCBP1l, LINC01224, SMC1B, ZNF541, HMX2, SPINK7 and KRT8P6. Our GO enrichment analysis of biological processes (BP) showed that the primary enriched terms included epidermis development, skin development, epidermal cell differentiation, keratinocyte differentiation and keratinization (Figure 2C). Notable cellular components included immunoglobulin complex, cornified envelope, intermediate filament, keratin filament, and circulating immunoglobulin complex (Figure 2D). The highlighted molecular function (MF) terms included antigen binding, activity of peptidase inhibitor, binding of immunoglobulin receptor, endopeptidase inhibitor activity, and structural constituents of the skin epidermis (Figure 2E). KEGG-related pathways included neuroactive ligand-receptor interactions, Staphylococcus aureus infection, IL-17 signaling, and xenobiotic and drug metabolism by cytochrome P450 (Figure 2F). These findings underscore the unique gene expression patterns that correlate with CSTF2 levels, indicating that these DEGs may be integral to the biological mechanisms involved in OSCC.

### 3.3 GSEA analysis of CSTF2 in OSCC

**Figure 3:**
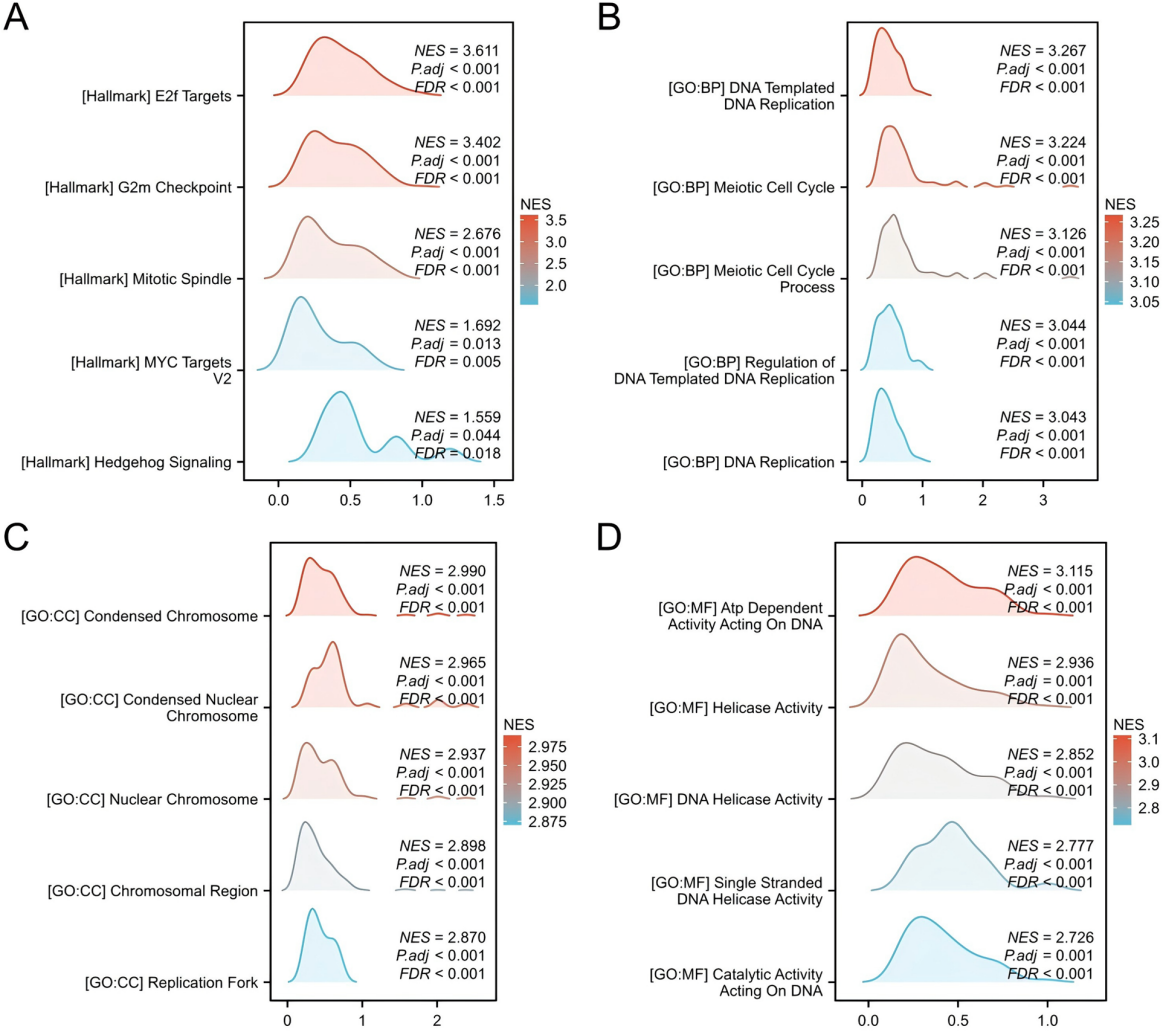
Gene set enrichment analysis (GSEA) of DEGs. GSEA enrichment results for **(A)** Hallmark genesets, **(B)** BP genesets, **(C)** CC genesets. **(D)** MF genesets.

Gene Set Enrichment Analysis (GSEA) was conducted to identify significant enrichment in diverse biological processes and signaling pathways. The results showed enrichment in processes hallmark datasets, including E2F targets, G2m checkpoints, Mitotic spindle, MYC targets V2 and Hedgehog Signaling. The Biological Processes section highlightes DNA-templated replication, meiotic cell cycle, and regulation of DNA replication. The cellular composition comprised condensed chromosomes, nuclear Chromosomes, chromosomal regions, and replication forks. Molecular functions included ATP-dependent activity on DNA, helicase activity, DNA helicase activity, single-stranded DNA helicase activity, and catalytic activity on DNA (Figure 3). Collectively, these results suggest a potential role for CSTF2 as a tumour promoter in OSCC through the activation of various signaling pathways.

### 3.4 Tumor immune signature analysis

**Figure 4:**
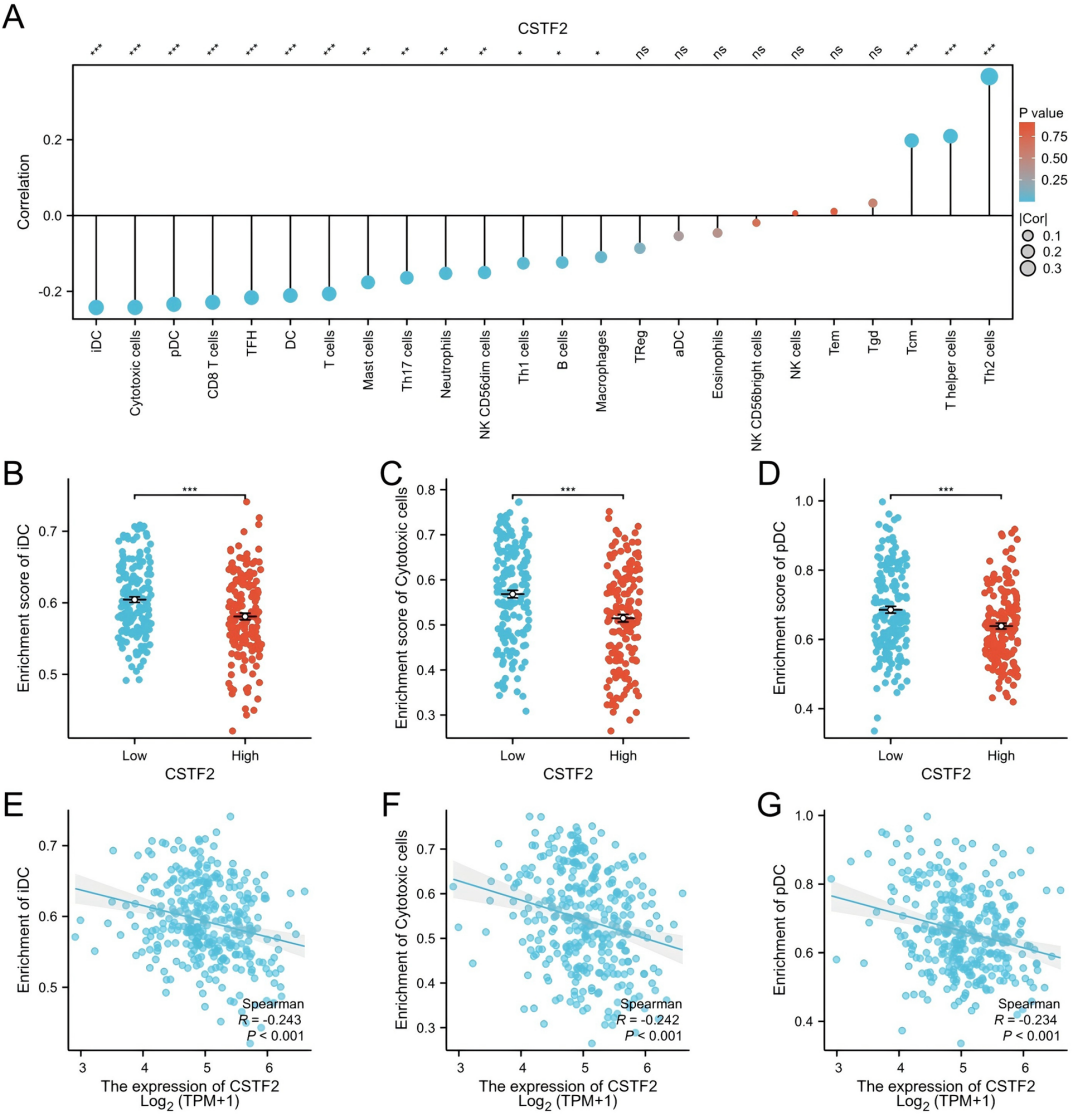
Correlation between CSTF2 expression and infiltration of immune cells in OSCC. **(A)** Overview of the correlation between CSTF2 expression level and different types of immune cells. **(B)** Impact of CSTF2 expression on iDC infiltration. **(C)** Impact of CSTF2 expression on Cytotoxic cell infiltration. **(D)** Connection between CSTF2 and infiltration of pDC cell. **(E)** Association between CSTF2 and iDC. **(F)** The correlation between CSTF2 and Cytotoxic cells. **(G)** Association between CSTF2 expression levels and plasmacytoid dendritic cell (pDC) infiltration.

The association between CSTF2 expression and the infiltration of diverse immune cell types was examined utilizing ssGSEA (Figure 4A). The analysis revealed a negative correlation between CSTF2 expression and the presence of immature dendritic cells (iDC), cytotoxic cells, and plasmacytoid dendritic cell (pDC) (Figures 4B–G). Conversely, a positive correlation was observed among Helper T cell 2 (Th2), T helper cells, and Central memory T cell (Tcm) (Supplementary Figures S2A–F). These results imply that CSTF2 may modulate the immune cell composition within the OSCC tumour microenvironment.

### 3.5 CSTF2 expression correlates with poor prognosis in OSCC

**Figure 5:**
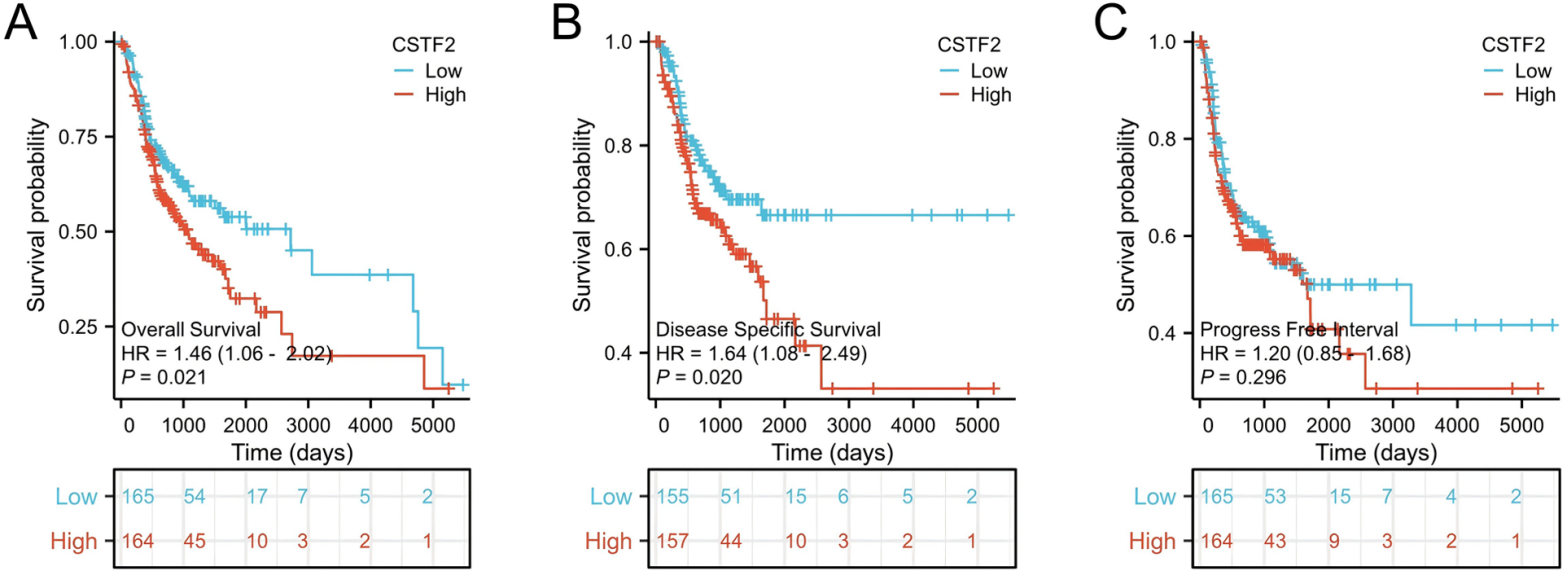
CSTF2 expression is linked to unfavorable survival in OSCC patients. **(A)** OS, **(B)** DSS. **(C)** PFI.

We conducted the Kaplan–Meier analysis to evaluated whether CSTF2 expression was correlated with the survival rates of OSCC patients. Our analysis showed that elevated CSTF2 expression was correlated with poor OS (*P* = 0.021) and DSS (*P* = 0.020) outcomes (Figures 5A,B). However, CSTF2 expression did not correlate with PFI (*P* = 0.296, Figure 5C). Additionally, we explored whether the overexpression of CSTF2 could assess patient survival in multiple cancer types other than OSCC. Elevated CSTF2 expression levels were markedly correlated with diminished OS in kidney renal clear cell carcinoma (KIRC), liver hepatocellular carcinoma (LIHC), head and neck squamous cell carcinoma (HNSC), pancreatic adenocarcinoma (PAAD), and sarcoma (SARC) (Supplementary Figure S2). Further ROC analysis indicated that PADD (AUC = 0.929) and SARC (AUC = 0.903) had higher AUC values than 0.7 (Supplementary Figure S3). Table 3 presents the Cox univariate and multivariate analyses of CSTF2 expression in OSCC patients. Multivariate analysis identified lymphovascular invasion (*P* < 0.001), radiation therapy (*P* = 0.002), and expression of CSTF2 expression (*P* = 0.043) as independent variables for OSCC prognosis.

**Table 3:** Cox univariate and multivariate analysis of OSCC patients.

Characteristics	Total	Univariate		Multivariate	
		Hazard ratio (95% CI)	*P*	Hazard ratio (95% CI)	*P*
Age	329	1.330 (0.961–1.840)	0.085	1.383 (0.864–2.215)	0.177
Gender	329	0.903 (0.645–1.266)	0.555		
Pathologic stage	298	4.075 (1.288–12.897)	**0** **.** **017**	0.331 (0.020–5.587)	0.443
Pathologic T stage	304	3.717 (1.592–8.676)	**0** **.** **002**	9.192 (0.866–97.557)	0.066
Pathologic N stage	275	2.222 (1.499–3.294)	**<0** **.** **001**	2.080 (0.967–4.475)	0.057
Alcohol history	321	1.039 (0.736–1.467)	0.827		
Histologic grade	321	1.681 (0.978–2.891)	0.060		
Lymphnode neck dissection	327	0.681 (0.449–1.033)	0.071	0.121 (0.036–0.410)	**<0.001** **
Lymphovascular invasion	239	1.711 (1.149–2.548)	**0**.**008**	1.719 (0.997–2.963)	0.051
Smoker	323	1.266 (0.857–1.870)	0.236		
Radiation therapy	294	0.601 (0.421–0.859)	**0**.**005**	0.427 (0.250–0.729)	**0**.002*
CSTF2	329	1.464 (1.059–2.024)	**0**.**021**	1.655 (1.017–2.694)	**0**.043*

* Statistically significant difference (*p* < 0.05).

** Statistically significant difference (*p* < 0.001).

### 3.6 The creation and validation of a nomogram for OSCC patient prognosis

**Figure 6:**
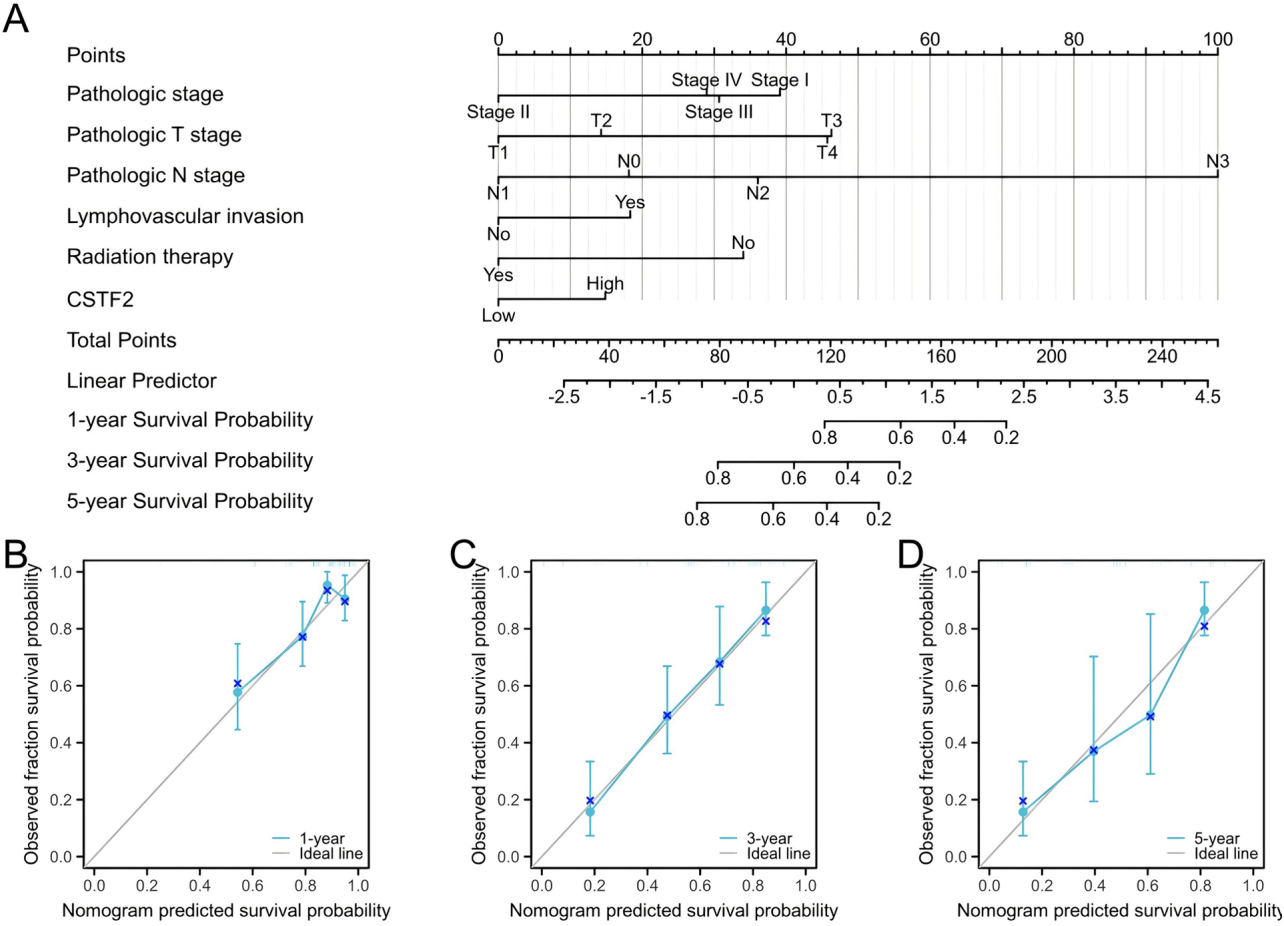
Prognostic diagnostic plots for predicting overall survival in OSCC patients. **(A)** A prognostic plot was created to forecast overall survival at intervals of 1, 3, 5 years. **(B)** Calibration curve demonstrating prediction accuracy for 1-year. **(C)** Prediction accuracy for 3-year. **(D)** Prediction accuracy for 5-year.

We developed a prognostic nomogram that incorporated independent prognostic factors to predict OS in OSCC patients. In the nomogram, elevated scores suggested worse prognosis (Figure 6A). Calibration curves were employed to assess the predictive accuracy of the nomogram (Figures 6B–D). The nomogram's bootstrap resampling consistency index (C-index) score was 0.734 (95% CI: 0.678–0.765), demonstrating a moderate level of accuracy in predicting overall survival rates of OSCC patient.

## 4 Discussion

As a prevalent malignancy in the oral cavity, OSCC substantially affects the quality of life and survival rates globally (18). In china, the incidence of OSCC is increasing, and is influenced by life style factors, such as cigarette smoking, alcohol abuse, betel quid and certain viral infections (19, 20). Recent data reveal an alarming increase in the incidence rate of OSCC within this area, coupled with a disturbing tendency for the disease to manifest at a younger age (21). Despite advancements in treatment approaches, including surgical intervention, radiotherapy, and chemotherapy, the prognosis of OSCC is unfavorable, primarily because of late-stage diagnoses, as symptoms are often subtle and nonspecific (22). Therefore, new molecular biomarkers are urgently needed to enhance the early detection and improve the prognostic assessments of patients with OSCC.

This study aimed to explore CSTF2 expression in OSCC tissues and its potential as a prognostic biomarker. Initially identified as a candidate oncogene in human T-cell lymphoma, high expression of CSTF2 has been observed in various cancers, including hepatocellular carcinoma, lung adenocarcinoma, and pancreatic ductal adenocarcinoma (23–26). However, its contribution to OSCC remains unclear. Using comprehensive bioinformatics analyses along with tissue microarray immunohistochemistry, our results suggest that CSTF2 expression is significantly high in OSCC. Furthermore, high CSTF2 expression was correlated with unfavorable clinicopathological factors, including patient age and gender. CSTF2 is known to promote cancer progression by affecting various signaling pathways such as c-Myc translation, IL-6/IL-6R, and Src/p190B (27, 28). However, CSTF2 mediated biological roles and signaling pathways require further investigation. In the current study, our GO/KEGG and GSEA analyses revealed that CSTF2 related expression group showed significant enrichment in pathways related to epidermal development and differentiation, immunoglobulin complex, peptidase and endopeptidase inhibitor activity, and cytochrome p450 metabolism. These findings requires additional experimental validation and could improve our understanding of CSTF2-related biological functions in OSCC.

Tumor cells exist within a complex microenvironment that includes not only cancer cells but also immune cells and various stromal components (29, 30). In malignant tumors, including OSCC, the tumor cells are typically surrounded by infiltrating immune cells (31). The prognostic impact of immune cell infiltration in solid tumors is determined by factors such as the immune cell type, density, and spatial distribution (32). Assessing infiltrating immune cells in OSCC may enhance ICI treatment and serve as a potential predictor of treatment outcomes (33). Notably, the elevated CSTF2 expression exhibited significant enrichment of the IL-17 signaling pathway. We examined the correlation between CSTF2 expression levels and the infiltration of various immune cells. Our study demonstrated that CSTF2 overexpression is inversely associated with the infiltration of iDC, cytotoxic cells, and pDCs, whereas it is positively associated with Th2 cells, T helper cells, and Tcm cells. These findings indicate that overexpression of CSTF2 could potentially affect the progression and prognosis of OSCC through the levels its modulation of infiltrating immune cell levels.

Prior studies have established an association between CSTF2 expression and decreased survival rates across various cancers (34, 35). Our survival analysis indicated that high CSTF2 expression independently predicted poor OS and DSS in OSCC patients. Therefore, CSTF2 may serve as a potential molecular biomarker of OSCC.

While the insights gained from this study are significant, it is crucial to recognize its limitations. The findings are primarily derived from assessments of data obtained from public databases, supplemented by immunohistochemical validation using tissue microarrays, which lack extensive validation through comprehensive clinical sample analyses. Furthermore, although we have proposed potential mechanisms that clarify the role of CSTF2 in OSCC, additional experimental studies are necessary to confirm the specific molecular pathways involved.

In conclusion, this study identified high CSTF2 expression as an independent negative prognostic indicator of OSCC, which was linked to aggressive clinical characteristics and immune regulatory mechanisms. CSTF2 may play a critical role in OSCC development through various signaling pathways. Nonetheless, the mechanism by which CSTF2 influences OSCC tumorigenesis and progression necessitate further investigation.

## Data Availability

The original contributions presented in the study are included in the article/Supplementary Material, further inquiries can be directed to the corresponding author.

## Supplementary material

Supplementary Figure S1 Positive correlation between CSTF2 expression and various immune cells. **(A)** Differential infiltration levels of Th2 cells in relation to CSTF2 expression. **(B)** T helper cells infiltration in relation to CSTF2 expression. **(C)** Tcm cell infiltration levels in relation to CSTF2 expression. **(D)** Correlation between CSTF2 and Th2 cell infiltration. **(E)** Relationship between CSTF2 and T helper cell infiltration. **(F)** Correlation between CSTF2 expression and Tcm cell infiltration.

Supplementary Figure S2 Prognostic values of CSTF2 expression in pan-cancer. **(A)** OS; **(B)** DSS; **(C)** PFI.

Supplementary Figure S3 ROC curves of CSTF2 in various cancer types. **(A)** HNSC; **(B)** KIRC; **(C)** PAAD; **(D)** LIHC; **(E)** SARC.

Supplementary Figure S4 Prognostic values of CSTF2 expression in patients with OSCC evaluated by the Kaplan–Meier method in different subgroups. **(A–K)** OS survival curves of Age >60 years; Male; Female; Pathologic stage III and IV; Pathologic stage T3 and T4; Pathologic stage N0 and N1; Histologic grade G1 and G2; Lymphovascular invasion, NO; Lymphnode neck dissection; Radiation therapy; Smoker and Alcohol history.
