# Supplementary figures and images for: Expression of CSTF2 in oral squamous cell carcinoma and its relationship with immune infiltration and poor prognosis
Source: Front Oral Health. 2025 Feb 7;6:1548829. doi: 10.3389/froh.2025.1548829 (PMC11842344; doi:10.3389/froh.2025.1548829)

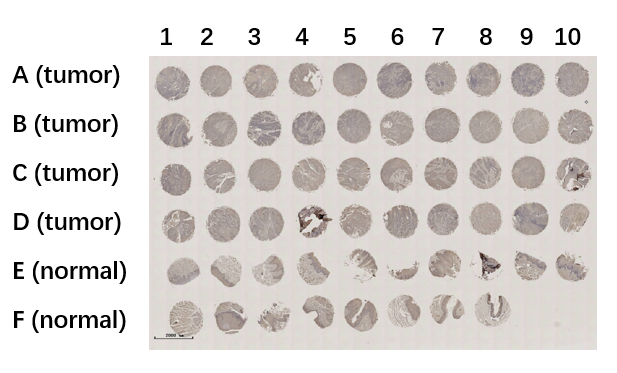

Supplement: Supplementary Figure S1 — Positive correlation between CSTF2 expression and various immune cells. (A) Differential infiltration levels of Th2 cells in relation to CSTF2 expression. (B) T helper cells infiltration in relation to CSTF2 expression. (C) Tcm cell infiltration levels in relation to CSTF2 expression. (D) Correlation between CSTF2 and Th2 cell infiltration. (E) Relationship between CSTF2 and T helper cell infiltration. (F) Correlation between CSTF2 expression and Tcm cell infiltration. [file Image1.png]

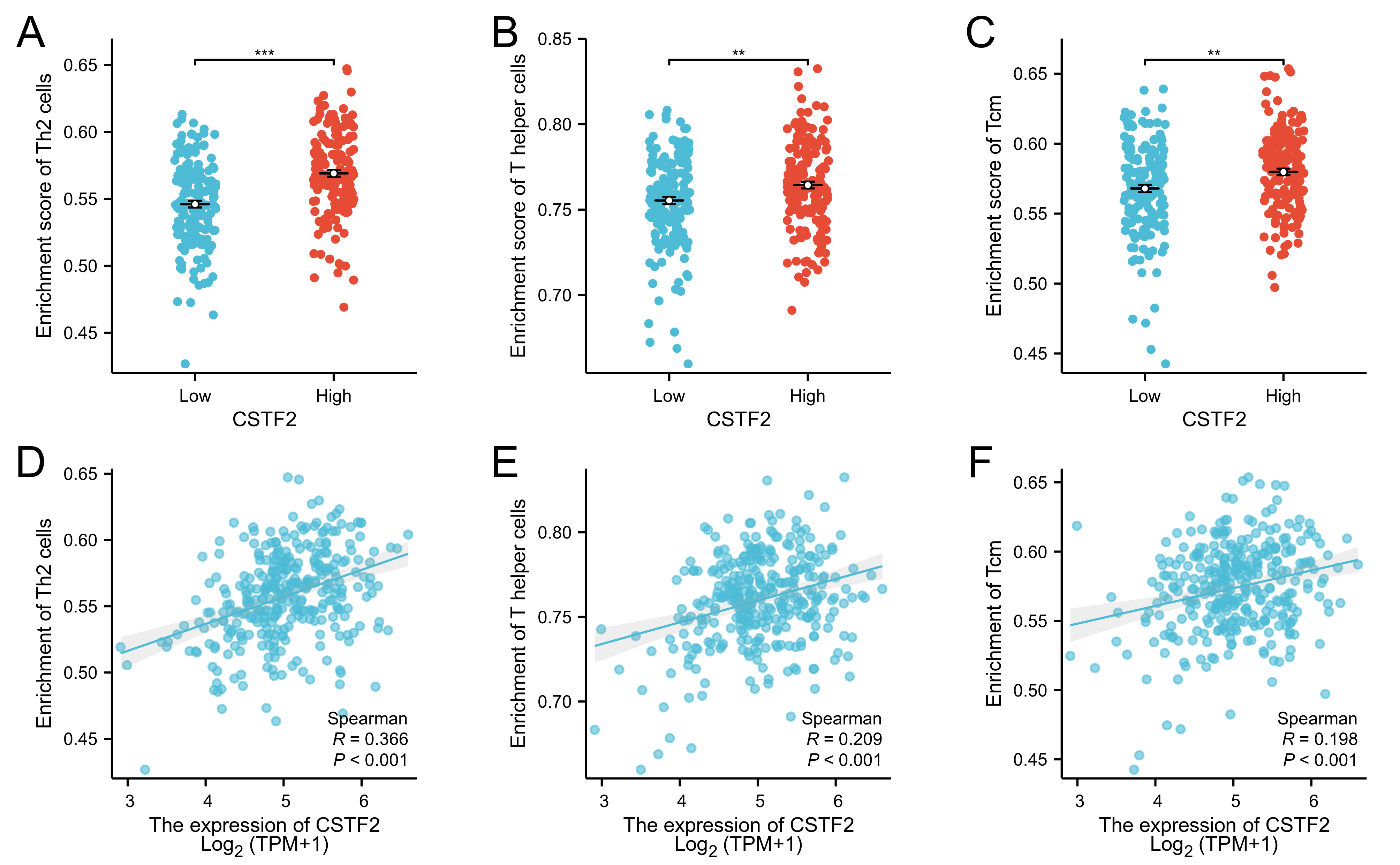

Supplement: Supplementary Figure S2 — Prognostic values of CSTF2 expression in pan-cancer. (A) OS; (B) DSS; (C) PFI. [file Image2.png]

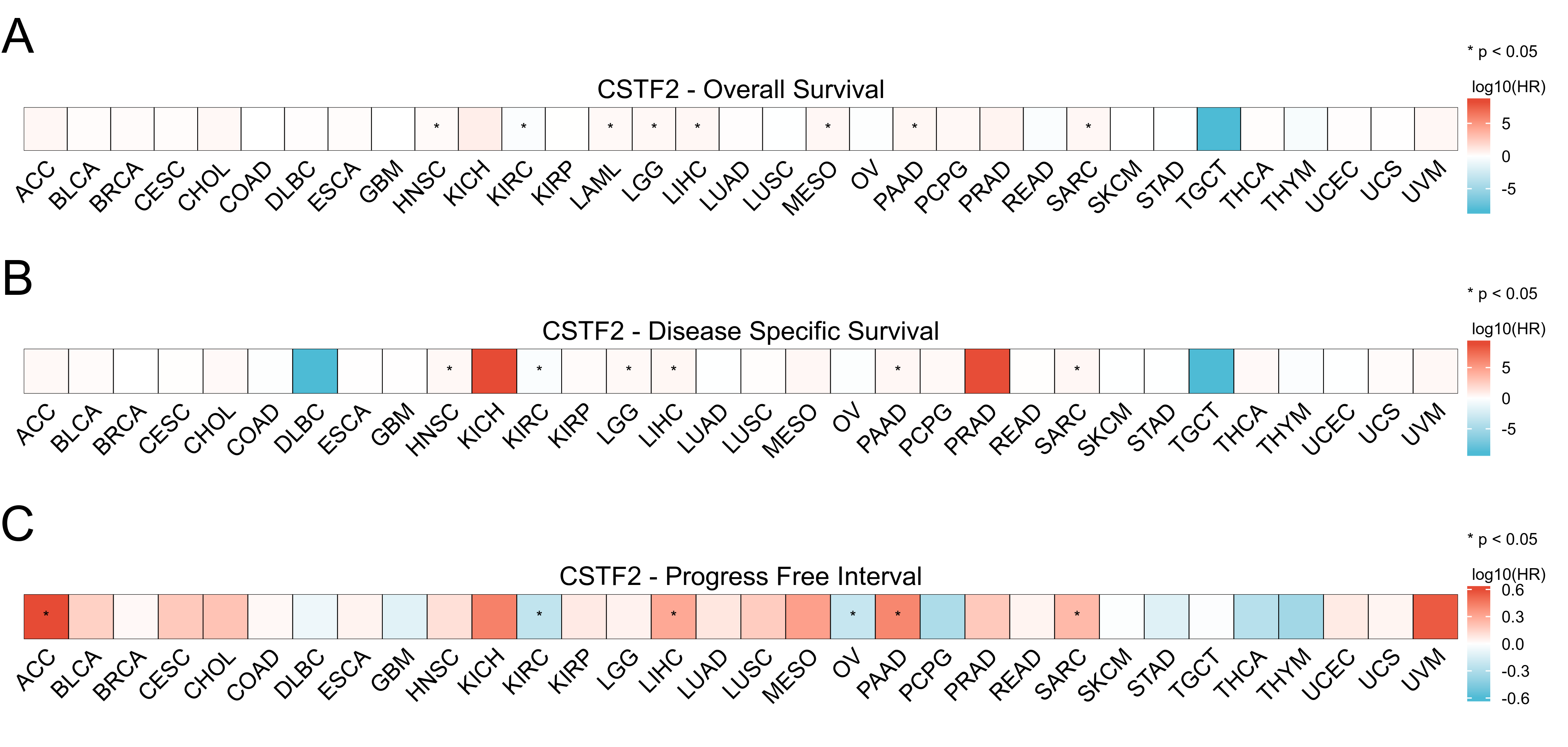

Supplement: Supplementary Figure S3 — ROC curves of CSTF2 in various cancer types. (A) HNSC; (B) KIRC; (C) PAAD; (D) LIHC; (E) SARC. [file Image3.png]

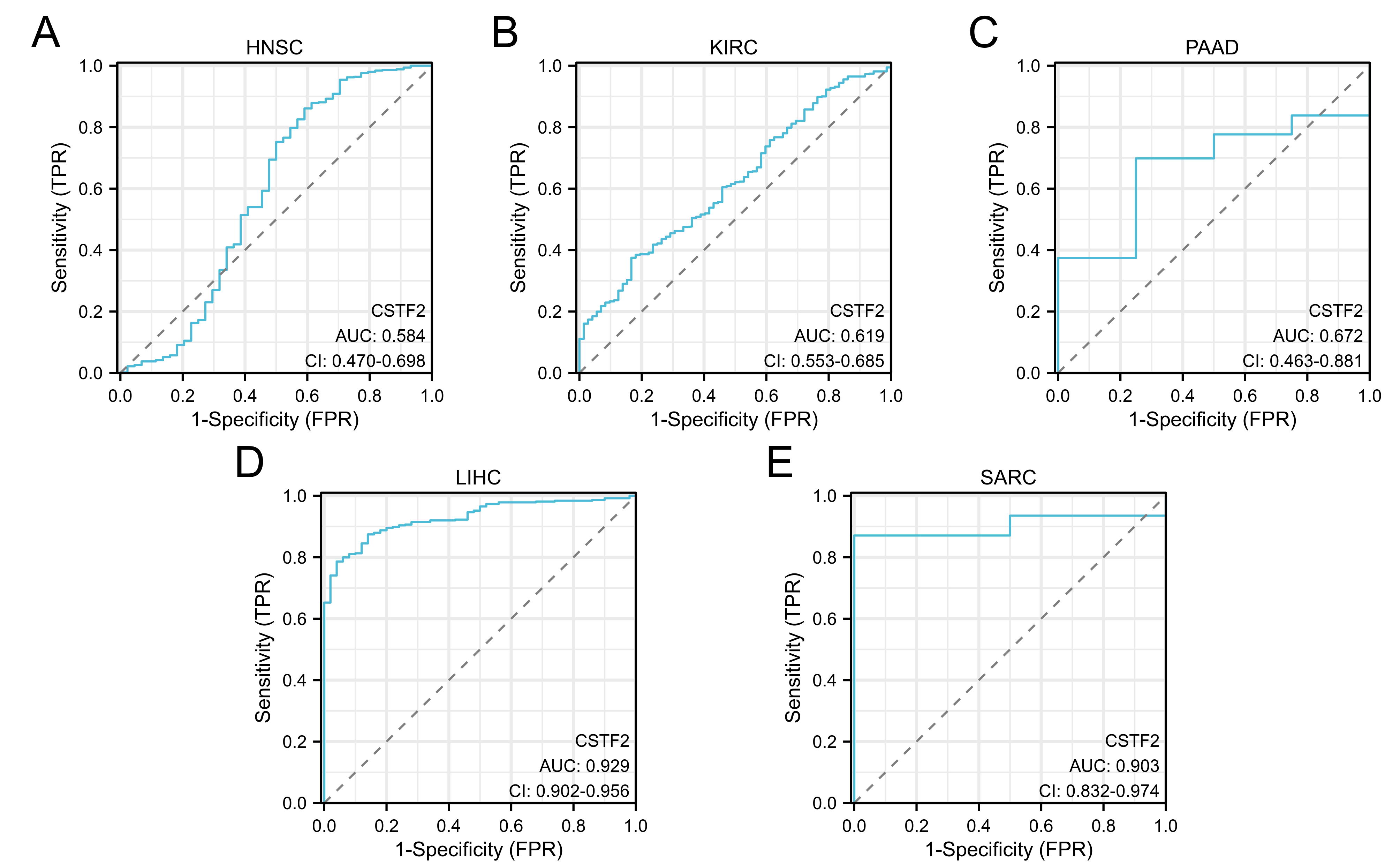

Supplement: Supplementary Figure S4 — Prognostic values of CSTF2 expression in patients with OSCC evaluated by the Kaplan–Meier method in different subgroups. (A–K) OS survival curves of Age >60 years; Male; Female; Pathologic stage III and IV; Pathologic stage T3 and T4; Pathologic stage N0 and N1; Histologic grade G1 and G2; Lymphovascular invasion, NO; Lymphnode neck dissection; Radiation therapy; Smoker and Alcohol history. [file Image4.png]

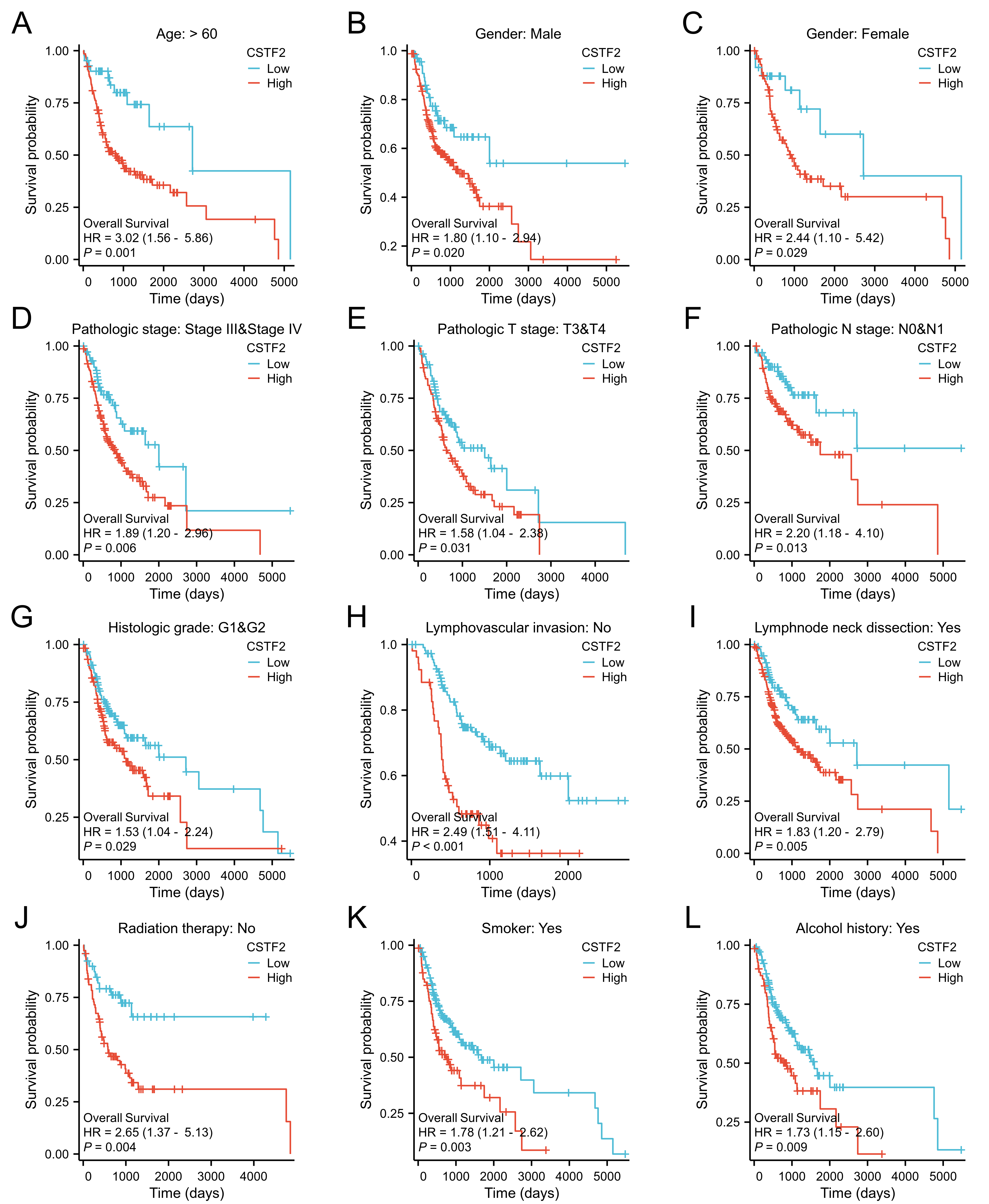

Supplement: Supplementary file 5 [file Image5.png]
